# DEAPR: Differential Expression and Pathway Ranking Tool Demonstrates *NRAS^G12V^* and *NRAS^G12D^* Mutations Have Differing Effects in THP-1 Cells

**DOI:** 10.3390/cancers17030467

**Published:** 2025-01-30

**Authors:** Susan K. Rathe, Jeremy P. White, Zohar Sachs, David A. Largaespada

**Affiliations:** 1 Masonic Cancer Center, University of Minnesota, Minneapolis, MN 55455, USA; 2 Division of Hematology, Oncology, and Transplantation, Department of Medicine, University of Minnesota, Minneapolis, MN 55455, USA; 3 Center for Genome Engineering, University of Minnesota, Minneapolis, MN 55455, USA; 4 Department of Pediatrics, University of Minnesota, Minneapolis, MN 55455, USA

**Keywords:** leukemia, RNA sequencing, *NRAS* mutations, calprotectin, differential expression, pathway analysis

## Abstract

**Background/Objectives**: *NRAS* mutations are found in approximately 10% of patients with acute myeloid leukemia (AML), with nearly half of those occurring at codon 12, but little is known about how differing G12 mutants affect cancer cell activity. **Methods**: A novel bioinformatic technique, differential expression and pathway ranking (DEAPR), was used to identify the most prominent changes in terms of both individual genes and associated pathways when comparing AML THP-1 cells containing an *NRAS^G12D^* mutation with B11 cells, which are THP-1-derived cells with the *NRAS^G12D^* allele removed and a dox-inducible *NRAS^G12V^* allele introduced. **Results**: In total, 1456 differentially expressed (DE) protein-coding genes were uniquely associated to the *NRAS^G12D^* mutation, while 585 DE protein-coding genes were specific to the *NRAS^G12V^* mutation. The innate immune system pathway was prominent in both mutant-specific lists, even though the genes involved were not in both lists. Furthermore, the two calprotectin genes (*S100A8* and *S100A9*), also associated with innate immunity, were upregulated in the *NRAS^G12D^* mutant and downregulated in the *NRAS^G12V^* mutant. **Conclusions**: This study, using the DEAPR strategy, clearly demonstrates the dramatic changes associated with two seemingly similar *NRAS* mutations, suggesting the deployment of different treatment strategies based on the type of *NRAS* mutation present.

## Simple Summary

*NRAS* proto-oncogene GTPase (*NRAS*) G12 mutations are generally considered to be activating mutations, but little is known about how they may differ. DEAPR, a novel approach for evaluating RNA-sequencing results, was used to identify the most prominent and reproducible changes to an AML cell line (THP-1) when the *NRAS^G12D^* mutation was replaced by an *NRAS^G12V^* mutation (B11 cells). The results demonstrate that the most common changes involved cell cycle activity. However, there was a dramatic difference in genes involved in the innate immune response, thus suggesting opportunities for *NRAS* G12 mutant-specific treatment options.

## 1. Introduction

Since its discovery in neuroblastomas in 1983 and its characterization as an oncogene [1], various mutations in *NRAS* have been identified in many types of cancer. An evaluation of 6876 cancer samples with *NRAS* mutations from the COSMIC database found that 23% had a G12 mutation [2]. In a study of 2502 patients with acute myeloid leukemia (AML), 112 were found to have *NRAS* mutations in codon 12, and of those 7 had a G12D mutation and 4 had a G12V mutation [3]. *NRAS* codon 12 mutations are considered activating mutations [4], and, indeed, the introduction of an *NRAS^G12V^* mutation into primary human melanocytes (PHM cells) resulted in the activation of the PI3K/AKC pathway [5]. In addition, *NRAS* mutations accompany the progression of preleukemic conditions to acute myeloid leukemia with poor prognosis [6]. Furthermore, a high-throughput drug screen tested the response of Ba/F3 cells to 843 drugs, when six different *NRAS* G12 mutations were introduced and showed varying results, indicating not all *NRAS* G12 mutations are the same [7], but more information is still needed on how different G12 mutants affect AML cells.

In previous work, the precise manipulation of *NRAS* was accomplished in THP-1 cells, a human AML cell line characterized by an *MLL-AF9* fusion and an *NRAS^G12D^* mutation [8]. The endogenous heterozygous NRAS^G12D^ mutation was removed using CRISPR and a dox-inducible *NRAS^G12V^* was introduced (B11 cells) to study the effects of *NRAS* mutations on self-renewal in human cells and compare them to our previous study of an *MLL-AF9/NRAS^G12V^* murine AML [9]. RNA-seq was performed on the parental THP-1 cells and the B11 cells with and without the presence of dox [8].

In the laboratory setting, the evaluation of the expression data from small experimental groups was problematic. When nine statistically based RNA-seq differential expression algorithms were compared, which included both parametric and non-parametric approaches, there were serious concerns raised in terms of power and false discovery rates when the sample sizes were *n* = 3 or *n* = 6 compared to *n* = 12. The low number of genes commonly identified by four of the better performing algorithms at the *n* = 6 versus *n* = 12 levels led to the conclusion that alternate methods were needed for evaluating smaller datasets [10].

DEAPR is one such method. It was developed to aid researchers in formulating hypotheses by identifying absolute differences in expression patterns to demonstrate the reproducibility of expression across small experimental sets, typically *n* = 3. It has proved to be a powerful tool for confirming the identity of samples, evaluating new experimental techniques, evaluating subgroups of Sleeping Beauty modified mouse samples, and in identifying the most prominent changes found in gene overexpression, knockdown, and knockout experiments, as well as drug assays [11].

DEAPR is a novel approach to weighing and ranking both genes and pathways, and it was used to compare the transcriptional changes when the G12D mutation was removed to those observed when the G12V mutation was expressed. DEAPR uses two novel methods for selecting differentially expressed (DE) genes, differential expression of low-variability (DELV) genes and separation of ranges by minimum and maximum (SRMM) values. DE genes are also screened to include only protein-coding genes, as well as absolute fold-change differences between the average fragments per kilobase of transcript per million mapped reads (FPKMs) of two or more for the DELV genes or the absolute SRMM of 2.0 or more for those genes failing to pass the DELV test but meeting the SRMM criteria. The selected genes are then ranked by both the absolute fold change (decreasing order) and absolute FPKM difference (decreasing order). A combined rank is then applied using 90% of the fold change rank and 10% of the FPKM difference rank. The resulting list of genes is input into GeneAnalytics [12] (geneanalytics.genecards.org) to generate a pathway list based on the PathCards unification database [13] (pathcards.gencards.org (accessed on 3 May 2023). GeneAnalytics uses a binomial distribution model to select the genes, and creates a score by correcting the *p*-value based on the false discovery rate. The resulting GeneAnalytics pathway list, sorted by the GeneAnalytics score, is adjusted by DEAPR based on the gene ranking of the genes associated with each pathway.

The tables and graphs generated from the DEAPR workflow provide a novel comparison of *NRAS* codon 12 mutants. The preponderance of the common pathways associated with both types of mutants revolved around cell cycle activity. In contrast, there was a dramatic increase in calprotectin expression in the G12D mutant and a comparable decrease in the G12V mutant, thus demonstrating how cancer cells can be highly sensitive to the amino acid substitution taking place at a single codon location.

## 2. Materials and Methods

### Analysis of Transcriptome Deep Sequencing Data (RNA-Seq)

HISAT2 v2.1.0 [6] was used to map the fastq data for samples S1–S18 (GSM3193270-SAM3193287), from a previously published experiment (GSE115911) [8]. StringTie v2.1.7 [14] was used to interrogate the HISAT2 output and generate expression levels presented in both fragments per kilobase of exon per million reads (FPKMs) and gene counts. The FPKMs and gene counts served as inputs to the DEAPR logic and DESeq2 v1.40.2 [15], respectively. The detailed specifications for the DEAPR workflow are provided in the Supplementary Methods. The ***deapr*** and ***pathway*** programs are available at https://github.com/LargaespadaLab/DEAPR (accessed on 3 May 2023).

## 3. Results

### 3.1. DEAPR Was Used to Identify and Rank Differentially Expressed Genes

To identify the DE genes associated with the *NRAS^G12D^* and *NRAS^G12V^* mutations, we used previously published RNA-seq data (GSE115911) from THP-1 cells, which is an AML cell line characterized by an *MLL-AF9* fusion and a heterozygous *NRAS^G12D^* mutation, and B11 cells, a THP-1-derived cell line with the *NRAS^G12D^* mutation replaced by a dox-inducible *NRAS^G12V^* mutation [8].

The samples were mapped using HISAT2, and the expression levels were calculated in FPKMs using StringTie. Six sets of samples with three replicates per set were used in the DEAPR comparisons. Three of the sets were THP-1 samples with 0, 1, and 10 μg of dox applied for 96 h prior to harvesting [8]. The other three sets were B11 cells, which also had dox introduced at 0, 1, and 10 μg levels. As previously shown, the B11 0 μg samples exhibited no evidence of the *NRA^SG12D^* mutation and only trace amounts of the *NRAS^G12V^* mutant, indicating minimal vector leakage, and the total FPKM levels for NRAS and the percentage of reads containing the mutation across all of the sets were comparable, except for the B11 cells with 10 μg of dox, which exhibited elevated levels of the *NRAS^G12V^* mutant allele [8]. As expected, NRAS protein levels were present at very low levels in the B11 cells in the absence of dox compared to the B11 cells with 1 μg or 10 μg dox [8].

**Figure 1:**
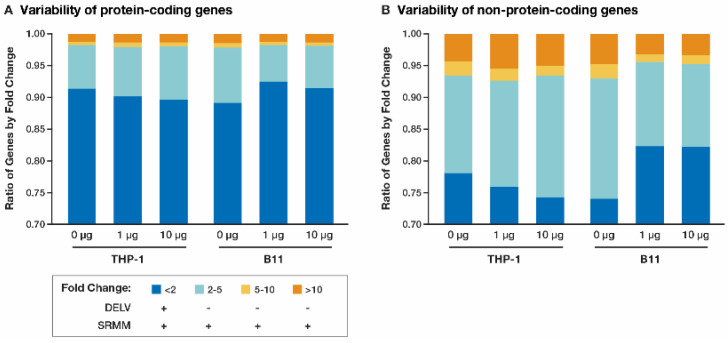
Frequency of the variability as measured by dividing the maximum FPKM value by the minimum FPKM value for each gene in each experimental set and subsetting by (**A**) protein-coding genes and (**B**) non-protein-coding genes.

The amount of variability in FPKM expression across the protein-coding genes within each experimental set was quite low, with genes undergoing less than a 2-fold change across the range in 89.0–92.5% of the genes (Figure 1A, Supplementary Table S1), indicating that the expression levels were reproducible and representative of each experimental condition. In contrast, non-protein-coding genes were highly variable, with genes having less than a 2-fold change across the range in 74.0–82.4% of the genes (Figure 1B, Supplementary Table S2), thus reflecting deficiencies in the mapping programs and transcriptome reference files when dealing with non-protein-coding genes.

**Figure 2:**
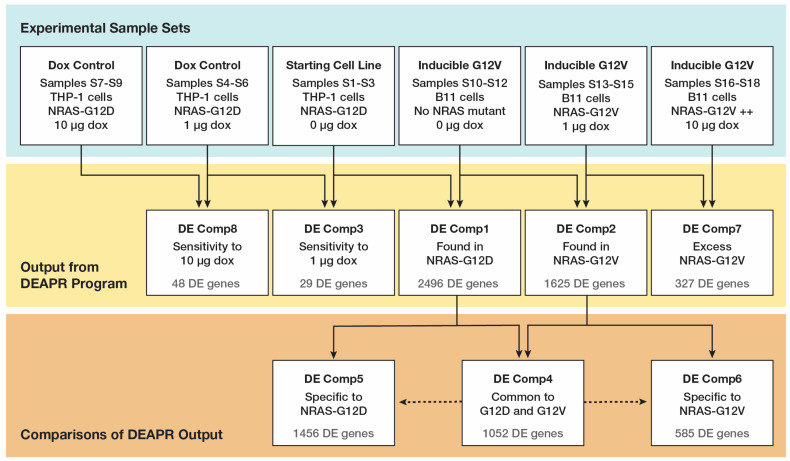
Schematic of comparisons conducted between the experimental sample sets (top row) with outputs from the comparisons using the ***deapr*** program (middle row) and outputs from the comparisons between the ***deapr*** outputs (bottom row). Dotted lines indicate exclusion from comparison.

Using the ***deapr*** program, five comparisons were performed (highlighted in yellow in Figure 2). Preliminary screening of the expression data selected protein-coding genes and excluded genes where the expression was less than 1 FPKM across all samples, since genes expressed at lower levels are highly variable, even between technical replicates [16]. Genes were considered DE genes if they met either of two requirements. The first requirement was differential expression of low variability (DELV), which first compared the highest and lowest FPKM values for each gene within a set of replicates to determine the fold change of the range and selected only genes having fold changes fewer than two in both sets of replicates being compared, thereby demonstrating reproducibility within each experimental set.

Then, the DELV logic compared the genes between the two sets looking for a fold change of two or more when comparing the average fold change between the two sets. Therefore, DELV identifies genes that are relatively invariant within a set of replicates but variable between compared sets. The second measurement was the separation of ranges by minimum and maximum (SRMM) values, which looked for genes with at least a 2-fold change between the minimum value in the one set and the maximum value in the other set. This two-pronged approach, using both DELV and SRMM values to identify DE genes, solved the dilemmas of (1) inadvertently selecting genes having significant overlap of expression values or (2) unwittingly excluding genes with no overlap and at least a 2-fold variance between experimental sets but containing a single outlier within one of the sets, which can be ignored by other methods because of their unusually high *p*-values and/or false discovery rates (FDRs), especially when evaluating small sets of data [10].

The parental cell line THP-1 0 μg dox (*NRAS^G12D^* mutant) was compared to the THP-1 1 μg dox and THP-1 10 μg dox cells, Comp3 and Comp8, with only 29 and 48 DE genes detected, respectively (Supplementary Tables S3 and S4) and most of those at low fold-change levels, indicating very little sensitivity to dox at either dose and, at the same time, demonstrating a low false discovery rate when using the DEAPR strategy. The B11 0 μg dox samples (no active *NRAS* mutant) were then compared to the THP-1 0 μg dox samples (*NRAS^G12D^*) and the B11 1 μg dox samples (*NRAS^G12V^*), which resulted in 2496 *NRAS^G12D^*-associated DE genes (Comp1) and 1625 *NRAS^G12V^*-associated DE genes (Comp2) being identified, respectively (Supplementary Tables S5 and S6). Of those DE genes, 1052 were common to both comparisons (Comp4, Supplementary Table S7), with 12 of the genes being dysregulated in opposite directions, so they were also included in the mutation-specific lists. This resulted in 1456 specific to the *NRAS^G12D^* (Comp5, Supplementary Table S8) and 585 DE genes specific to the *NRAS^G12V^* mutants (Comp6, Supplementary Table S9). The increased level of doxycycline exposure to the B11 *NRAS^G12V^* cells (Comp7, Supplementary Table S10) generated an additional 327 DE genes. There were only seven DE genes common to 1 μg dox in both the THP-1 cells and the B11 cells (Comp3 and Comp2), and only one with an expression change in the same direction, again indicating a low likelihood that the cells experienced changes specific to the doxycycline exposure.

In each of the comparisons, the genes were weighted and ranked using a novel approach that considers both the fold change and FPKM levels. Theoretically, if two DE genes have similar fold changes but one of the DE genes was expressed at much higher levels than the other, the DE gene with the higher expression should have a greater impact on cellular behavior due to a variety of mechanisms [17]. Therefore, for each comparison, the genes were independently ranked by fold change and FPKM difference. Then, a combined rank was applied using 90% of the fold change rank and 10% of the FPKM difference rank.

### 3.2. A Comparison to the DESeq2 Results Demonstrates the Utility of DEAPR

DESeq2, a widely used differential expression technique designed to use normalized gene counts rather than FPKMs, was executed to generate comparable Comp1 results. Using standard practices, DE genes were selected from the DESeq2 output by selecting genes with an adjusted *p*-value < 0.05 and then sorting the genes by absolute fold change from highest to lowest. DESeq2 identified 5022 total DE genes, with 3602 being protein-coding, while DEAPR, which by design selects only protein-coding genes, identified 2496 DE genes. Of the 3602 DESeq2 protein-coding DE genes, 2200 were also identified by DEAPR.

To better understand the differences between the DESeq2 and DEAPR lists of DE protein-coding genes, a detailed analysis was performed on the top 500 DE genes from each list (Supplementary Tables S11 and S12).

**Figure 3:**
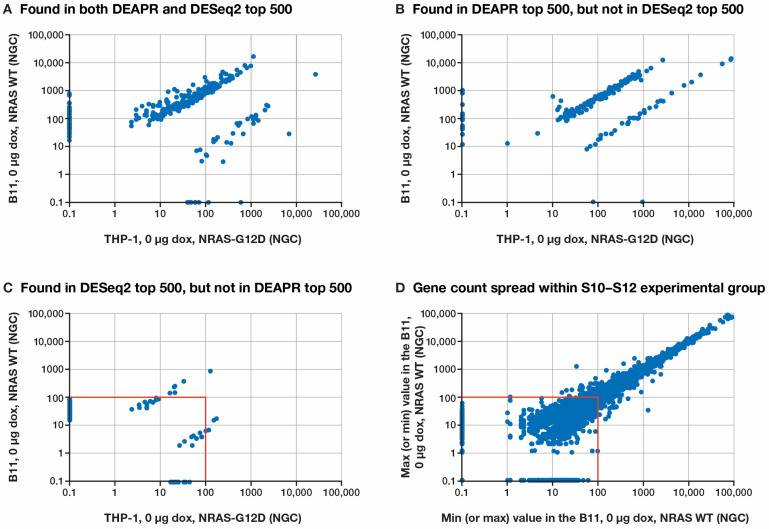
A comparison of the normalized gene counts (NGCs) between the top 500 ranked DE genes for the Comp1 comparison as identified by DESeq2 and the top 500 DE genes identified by DEAPR, using the normalized gene counts from the DESeq2 analysis: (**A**) 312 common to both top 500 lists; (**B**) 188 genes in the top 500 DEAPR list but not the top 500 DESeq2 list; (**C**) 188 genes in the top 500 DESeq2 list but not the top 500 DEAPR list; (**D**) gene count spread within the S10–S12 experimental control group. The red square denotes an area of high variability. (NGCs provided in log scale, with the zero gene count values set to 0.1).

There were 312 DE genes common to both lists (Figure 3A). Of the 188 genes identified as among the top 500 of the DESeq2 list but not among the top 500 of the DEAPR list (Figure 3C), DEAPR ignored 173 genes not meeting the minimum requirements for either the FPKM or SRMM values, while 15 genes appearing in the DEAPR list had ranks of 501+ in the DESeq2 list (Supplementary Table S11). Of the 188 genes identified among the top 500 of the DEAPR list but not among the top 500 of the DESeq2 list (Figure 3B), DEAPR identified 9 genes that were excluded from the DESeq2 list due to high or missing FDRs and 179 genes that were not in the top 500 of the DESeq2 list (Supplementary Table S12). Most of the genes ignored by DEAPR appeared in the lower left quadrant in the Figure 3C graph, in an area of high variability within the S10-S12 experimental set (Figure 3D), which was used as the *NRAS* WT control in the Comp1 comparison.

Of the top 500 DESeq2 genes that did not show up among the top 500 of the DEAPR analysis, there was a demonstratable bias toward longer gene transcripts that was not seen in the DEAPR-only genes (Supplementary Figure S1A–C). Furthermore, an examination of the top 50 genes from both the DESeq2 and DEAPR ranking methods showed a bias by DESeq2 toward genes expressed at lower levels (Supplementary Figure S2A,B), which is consistent with the results seen for the top 500 (Figure 3C).

When the top 20 genes from the Comp1 DEAPR list and the top 20 genes from the Comp1 DESeq2 list were examined more closely (Supplementary Tables S13–S16), some interesting patterns emerged.

As expected, the FPKM levels and gene count levels were highly comparable for each individual gene, thereby eliminating the use of different measurements as a factor when comparing the two methodologies. On the contrary, the primary differences between the DEAPR and DESeq2 approaches, which ultimately affected the rankings, was in how each method handled zero values and calculated the fold change.

DESeq2 does not establish a minimum detectable level or a minimum difference between groups. Its methodology modifies the fold change based on the dispersion values. DEAPR establishes a minimum detectable FPKM of 0.01 to use in fold change calculations. It uses the SRMM calculation to determine the minimum fold change and minimum FPKM difference in cases where there is a high degree of variability within an experimental group; otherwise, for the DELV (i.e., low-variability) genes, it uses the averages of each experimental group to establish the fold change and FPKM difference.

A prime example of how these differences affected the rankings is the 7th-ranked gene (*MMP9*) in the DEAPR list (Supplementary Table S13). *MMP9* was ranked as the 2046th gene in the DESeq2 list and yet was obviously highly upregulated when the NRAS^G12D^ mutant was present (Supplementary Figure S3).

After a careful examination of the genes exhibiting this type of behavior, it was theorized the cause stemmed from a gene having one or two zero counts in one of the experimental groups, as was the case with *MMP9*, which had 18 gene counts in sample S10, while samples S11 and S12 had 0 gene counts. To test this theory, a single gene count was added to all genes in all samples, and DESeq2 was run again. This resulted in DESeq2 assigning ranks to these genes that were comparable to the DEAPR rankings, with *MMP9* moving from 2046th to 15th, *TCEA3* from non-ranked to 22nd, and *AP0019311.1* from 886th to 3rd. But this had the unfortunate side effect of increasing DESeq2’s bias toward genes expressed at lower levels, which resulted in pushing many genes not only out of the top 100 but out of the top 500 as well (Supplementary Figure S4A,B and Supplementary Table S17).

A summary of the primary differences between DEAPR and DESeq2 is presented in Table 1.

### 3.3. Both NRAS^G12D^ and NRAS^G12V^ Mutant Cells Have Increased Cell Cycle Activity Compared to Controls

The top 400 ranked DE genes identified by DEAPR were submitted to GeneAnalytics (GA) and the score from the GA pathway tool modified based on the gene ranking (***pathway*** program). The choice of 400 DE genes is discussed in the Supplementary Methods document. Since the B11 cells with no dox represent THP-1 cells with no-NRAS mutation, B11 no-dox cells were compared to both the starting THP-1 cells with 0 dox having the heterozygous *NRAS^G12D^* mutation (Comp1) and the B11 cells with 1 μg dox having the het *NRAS^G12V^* mutation at comparable levels (Comp2). The results of the DEAPR modified GA pathway analysis for Comp1 (*NRAS^G12D^*) and for Comp2 (*NRAS^G12V^*) independently showed that the top 10 pathways in both comparisons were the same, albeit in slightly different orders, and most of the pathways were associated with cell cycle activity (Supplementary Tables S18–S20).

The increased cell cycle activity of *NRAS^G12D^* and *NRAS^G12V^* samples versus controls was also evident in previously published results of CyTOF experiments and colony-forming assays for these same cell lines [8].

### 3.4. NRAS^G12D^ and NRAS^G12V^ Mutant Cells Have Mutation-Specific Expression Patterns

To delve deeper into the mutant-specific expression changes, the common genes that increased or decreased in both *NRAS^G12D^* and *NRAS^G12V^* from the Comp4 comparison were removed from the Comp1 and Comp2 lists to create an *NRAS^G12D^*-specific list (Comp5) and an *NRAS^G12V^*-specific list (Comp6), respectively.

**Figure 4:**
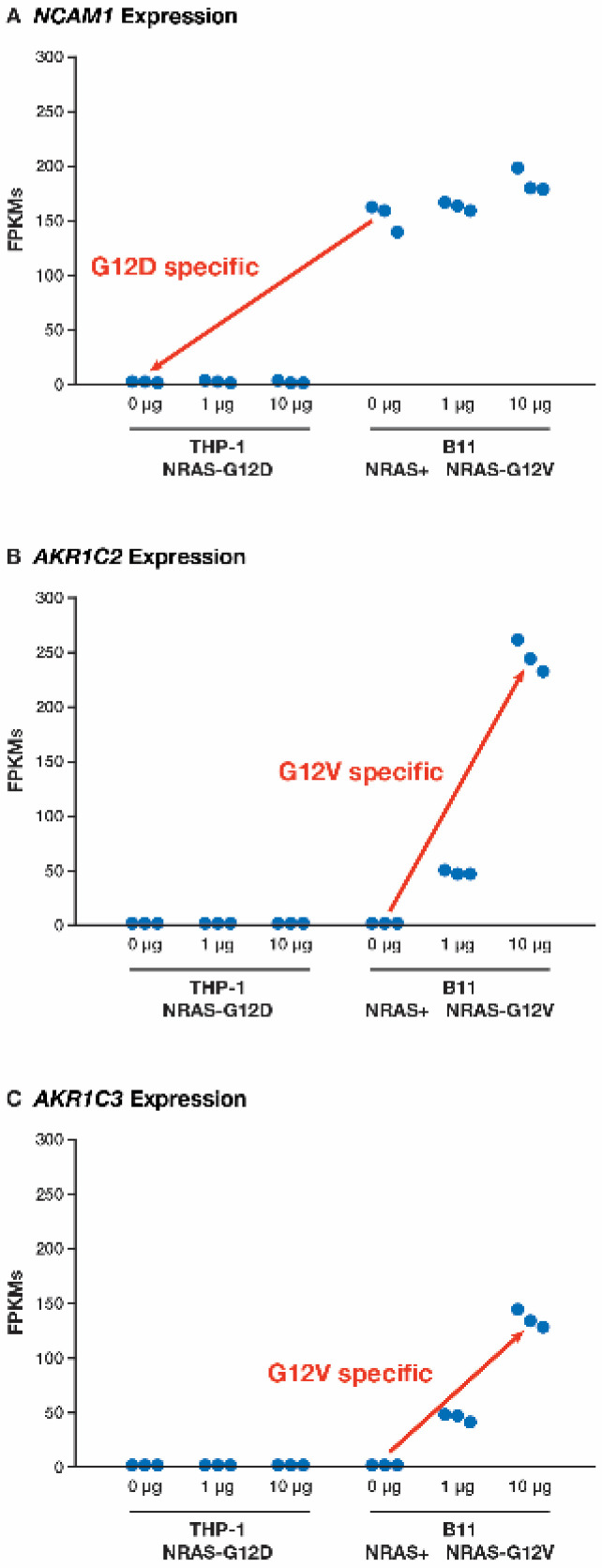
The FPKM levels of specific genes for each sample, organized by experimental set. (**A**) For the *NCAM1* gene, the red arrow indicates the comparison between the no-*NRAS*-mutant control (B11 0 μg dox) and the *NRAS^G12D^* mutant (THP-1 0 μg dox), indicating that the lack of *NCAM1* expression is specific to the *NRAS^G12D^* mutant. For the (**B**) *AKR1C2* gene and (**C**) *AKR1C3* gene, the red arrows indicates the comparison between the no-*NRAS*-mutant control (B11 0 μg dox) and the *NRAS^G12V^* mutant (THP-1 with both 1 and 10 μg dox), indicating that the overexpression of *AKR1C2* and *AKR1C3* is specific to the *NRAS^G12V^* mutant and dose-dependent (*n* = 3 in each set of samples).

The most highly ranked dysregulated gene in the *NRAS^G12D^*-specific list was *NCAM1*, which was not expressed in the THP-1 cells but had high expression in the B11 0 μg dox (no-*NRAS* mutant) and retained high levels when dox was introduced into the B11 cells to activate the *NRAS^G12V^* mutant (Figure 4A, Supplementary Table S8). The *CD56* isoform of *NCAM1* is expressed in 20% of patients with AML and has been found to play important roles in cell survival and stress resistance [18,19]. Although many isoforms have been identified for *NCAM1*, *CD56* is nearly identical to the canonical isoform, except for the skipping of exon 9, which results in the loss of 10 amino acids. In the B11 samples in this study, ~30% of the reads skipped exon 9. As for the *NRAS^G12V^*-specific list, two of the most highly ranked dysregulated genes were *AKR1C2* and *AKR1C3* (Figure 4B,C, Supplementary Table S9), with both appearing to be dose-dependent.

An analysis of the pathways associated specifically with the *NRAS^G12D^* or *NRAS^G12V^* mutant samples resulted in the exclusion of common cell cycle activity genes while magnifying the importance of other pathways. Surprisingly, both the G12D list and the G12V list of the top 10 pathways (Supplementary Tables S21–S23) included both the innate immune system pathway and the signal transduction pathway with the highest weighted GA scores, even though all common genes with expression changes in the same direction had been removed from both lists.

The three pathways ranked ahead of the innate immune system pathway in the *NRAS^G12D^*-specific list shared a common gene, *SPP1*, implicating it as a major player in the *NRAS^G12D^* phenotype (Supplementary Methods, Supplementary Figure S5). High expression of *SPP1* (osteopontin) has been found in dormant leukemia-initiating cells and has been suggested as a potential therapeutic target [20].

### 3.5. Calprotectin Levels Are Upregulated by NRAS^G12D^ and Downregulated by NRAS^G12V^

**Figure 5:**
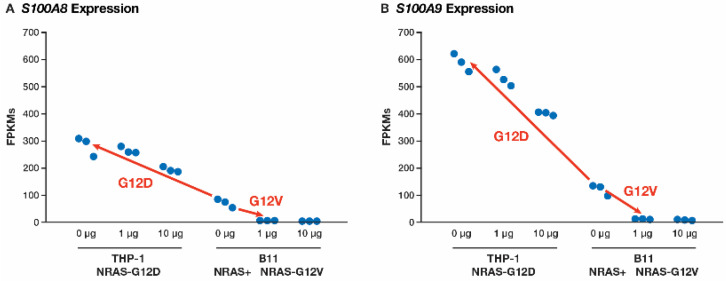
FPKM levels for the (**A**) *S100A8* and (**B**) *S100A9* genes for each sample, organized by experimental set. For both genes, the red arrows indicate a comparison between the no-*NRAS*-mutant control (B11 0 μg dox) and both the *NRAS^G12D^* mutant (THP-1 0 μg dox) and NRAS^G12V^ mutant (THP-1 1 μg dox). Both genes were significantly upregulated in the *NRAS^G12D^* mutant and significantly downregulated in the *NRAS^G12V^* mutant (*n* = 3 in each set of samples).

In the list of 1052 DE genes common to both mutations (Comp4), there were 12 DE genes for which the expression changed to the opposite direction. The top two genes displaying this phenomenon were *S100A9* and *S100A8*, which were ranked 106th and 175th, respectively. In the *NRAS^G12D^*-specific list (comp5, Supplementary Table S8), *S100A9* was ranked 109th and *S100A8* was ranked 180th, while in the *NRAS^G12V^*-specific list (comp6, Supplementary Table S9) *S100A8* was ranked 4th and *S100A9* was ranked 7th. *S100A8* and *S100A9* form the heterodimer calprotectin. *S100A8* and *S100A9* had SRMM fold changes of 4.1 and 5.0, respectively, when comparing the G12D samples (THP-1, no dox) to the no-mutation samples (B11, no dox), whereas *S100A8* and *S100A9* had an SRMM fold change of −23.5 and −12.8, respectively, when compared the G12V samples (B11, 1 μg dox) to the no-mutation samples (B11, no dox) (Figure 5A,B).

Of the 12 genes appearing to be dysregulated in opposite directions, 7 of the genes were associated with the innate immune system pathway (*S100A8*, *S100A9*, *CD209*, *IL31RA*, *C3*, *XAF1*, and *TNFSF14*), but only *S100A8* and *S100A9* appeared in the top 400 ranked DE genes used in the pathway analysis.

### 3.6. Elevated Expression of NRAS^G12V^ in B11 Cells Increases EGR1 and TERT Expression While Decreasing Expression of S100A

**Figure 6:**
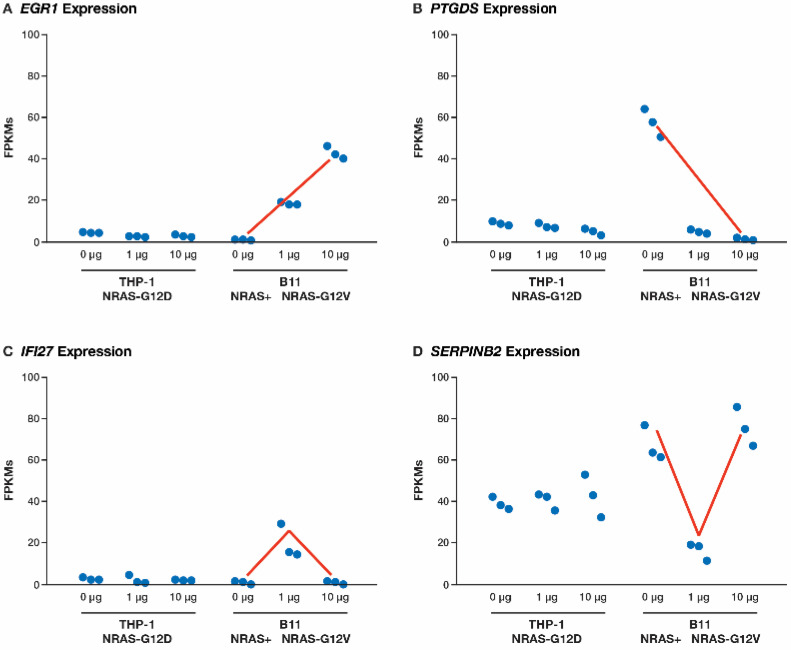
FPKM levels for representative genes. Each sample is organized by experimental set and shows the dose-dependent responses to dox in the B11 cells. The red lines indicate the following: (**A**) an upward trend in the *EGR1* expression; (**B**) a downward trend in the *PTGDS* expression; (**C**) an A-shift pattern in the *IFI27* expression; (**D**) a V-shift pattern in the *SERPINB2* expression (*n* = 3 in each set of samples).

Some interesting patterns emerged when elevating the levels of *NRAS^G12V^* by increasing dox from 1 μg to 10 μg (Supplementary Table S24). Of the 141 DE genes selected by DEAPR in this comparison (Comp7), there were 15 genes showing a significantly increased upward trend (Figure 6A), including four oncogenes (*AKR1C2*, *CCND1*, *EGR1*, and *PIR*), *TERT*, and a gene associated with doxorubicin resistance, *AKR1C3* [21]. There were 36 genes with a downward trend with the top-ranked gene in this category being *PTGDS* (Figure 6B). There were 78 genes, well over half of the genes, showing an A-shift pattern (Figure 6C). This same A-shift pattern was found in several proteins evaluated by CyTOF from this same set of samples when looking for dose-dependent responses 4, and, finally, there were 11 genes with a V-shift pattern (Figure 6D).

*SERPINB2*, a senescence marker capable of binding and stabilizing *p21Cip1* encoded by *CDKN1A* [22], was the top-ranking gene with the V-shift pattern. It is not surprising that *SERPINB2* increased as the cell cycle activity decreased following the removal of the *NRAS^G12D^* mutation (B11 0 μg samples), and it decreased back to *NRAS^G12D^* levels upon introduction of the *NRAS^G12V^* mutation. However, the dramatic increase in *SERPINB2* in the elevated *NRAS^G12V^* (B11 10 μg) samples was unexpected, especially since there were no apparent changes in the cell cycle activity based on the pathway results, which was also expected with the reduction in *p21Cip1*’s inhibitory activity. It is also noteworthy that over 80% of the dysregulated genes (115 of 141) were downregulated with the increase in the dox dosage, suggesting the presence of powerful feedback loops at this increased level of *NRAS^G12V^* expression. Furthermore, according to the GeneAnalytics output, 34% of the downregulated genes (39 of 115) were associated with the innate immune system, including *S100A8*, implicating a further change in the immune response associated with increased *NRAS^G12V^*.

## 4. Discussion

In general, when comparing RNA-seq to high-throughput qPCR techniques, RNA-seq is deficient when evaluating genes expressed at low levels, while qPCR struggles with genes that generate multiple isoforms. Despite these differences, there is a moderately high correlation (~85%) between the two methods when comparing the relative expression level changes of the protein-coding genes [23]. In laboratory experiments in which cells are exposed to extreme conditions to identify the most dramatically affected genes, RNA-seq offers a more inclusive method to determine the genes most affected by the experimental conditions. Cancer researchers can benefit greatly from the RNA-seq of small sets of experimental data if there is a method available to select genes specifically demonstrating reproducibility while ignoring genes expressed at such low levels as to be outside the sensitivity of the RNA-seq approach.

**Figure 7:**
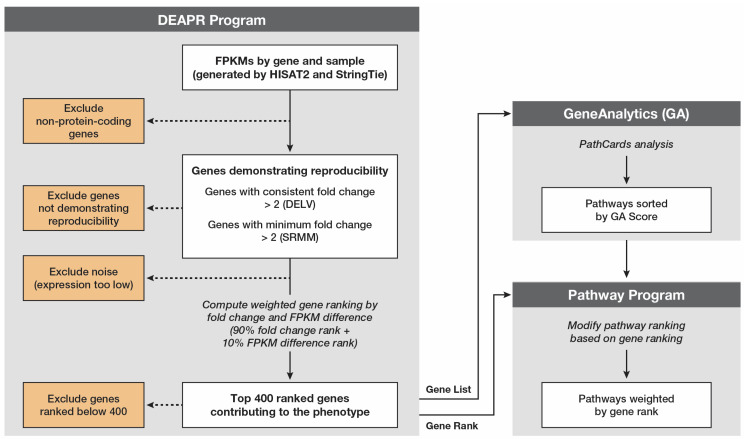
Schematic of the DEAPR workflow, which was used to compare the two experimental groups. DEAPR consists of three parts (grey boxes), with the data represented by white boxes and exclusionary logic by orange boxes.

To demonstrate this, DEAPR logic (Figure 7) was used to evaluate the differences between the AML cell lines when an *NRAS^G12D^* mutation was replaced by an *NRAS^G12V^* mutation. One of the biggest challenges for researchers using RNA-seq data to evaluate experimentally modified samples was identifying the most prominent changes taking place when working with small sets of data, as can be seen by the lack of overlap in the DE genes identified by a comparison of nine DE methods [10] and the requirement of more samples than is practical. Ranking the DE genes is also challenging. Most frequently, the fold change is used to rank DE genes, but this can ignore the amplification effects of genes expressed at high levels. But, on the other hand, using the FPKM difference alone diminishes the importance of highly potent oncogenes when expressed at low FPKM levels. Only by combining the fold change and FPKM difference can a true picture of a gene’s impact on cellular behavior be appreciated.

Another deficiency of other DE algorithms is their treatment of outliers, which can result in a gene being ignored entirely because of high FDRs or downplayed in their importance, as was seen with *MMP9* in the Comp1 analysis comparing the DEAPR with the DESeq2 results. Cells can be quite sensitive to the timing of the experiment, as was seen when the B11 cells were exposed to an increased dose of dox, causing 80% of the dysregulated genes to become downregulated. Rather than ignoring genes that are experiencing feedback modulation effects that distort FDRs, the SRMM logic provides a minimum fold change view by comparing the lowest value in one set of samples to the maximum value in another set of samples. In the case of *MMP9*, it would have been overlooked as a key player in *NRAS^G12D^* mutant-driven AML progression if using DESeq2, whereas DEAPR highlighted its role by placing it in the top 10 genes. Since *MMP9* is a well-known contributor to AML progression, as well as a disease marker and therapeutic target [24], its placement by DEAPR appears to be the logical interpretation.

The DESeq2 results also showed a strong bias toward longer genes, which is associated with the use of normalized gene counts. An additional issue with using DESeq2 stems from the lack of a filter for the minimum reliable level of expression, which is admittedly difficult to do with the gene count normalization method used by DESeq2, since it does not include gene length as a factor. The use of an FPKM of 1.0 as a minimum reliable level of expression in DEAPR was shown to be appropriate for the depth of the samples involved. Since FPKM includes gene length as a factor, there is no bias based on the gene length. Furthermore, since the hijacking of the transcriptional machinery has been demonstrated by both oncogenic [25] and viral [26] factors, it is not surprising to see how the overexpression of genes in an experimental setting can lead to a high degree of variability in genes expressed at lower levels, as was shown in Figure 3D. The inclusion of these questionable DE genes identified by DESeq2 could have a dramatic effect on subsequent pathway analyses.

The selection of the PathCards database to identify the most prominent pathways was the culmination of years of experience with various pathway databases and tools, most of which supply multiple answers based on the database being used. PathCards combines data from a dozen different databases and applies a SuperPath designation. GeneAnalytics uses an over-representation analysis (null hypothesis) approach to interrogate the PathCards database and applies a score to each pathway for ranking. This score is easily modifiable by applying weights based on the gene ranking, as is accomplished by the last step of DEAPR (the *pathway* program). From a practical perspective, at least 100 DE genes need to be included in the list provided to GeneAnalytics to generate an informative list of pathways associated with the comparison being performed. However, using more than 400 DE genes can cause some pathways that have a low number of genes associated with them to be excluded from the analysis, as was demonstrated here.

In fact, GeneAnalytics warns in their user guide of a potential bias toward pathways containing more genes, if more than 300 DE genes are input, but that is assuming all 300 genes are associated with at least one pathway. This is also a potential danger when using other pathway analysis tools, but by using the DEAPR approach to rank and select the top DE genes, the most impactful genes associated with the phenotype being analyzed will be used to generate and prioritize the pathway list. The use of GeneAnalytics as a component of the DEAPR logic effectively identified the increase in cell cycle activity when either *NRAS^G12D^* and *NRAS^G12V^* was present, which is consistent with the CyTOF and colony-forming experiments previously performed on the same cell lines [8].

The DEAPR analysis highlighted many genes that were already being studied in association with AML and which had been suggested as therapeutic targets, such as *MMP9* [24] and *NCAM1* [27]. The DEAPR weighting strategy not only emphasized the importance of *MMP9* in the *NRAS^G12D^* phenotype, but it also suggested *MMP9* expression was mutation-specific. In the case of *NCAM1*, all isoforms of *NCAM1* were suppressed in the *NRAS^G12D^* samples, but this suppression was lost when the *NRAS^G12D^* mutation was removed, indicating that the *NCAM1*-targeted treatments may be less effective in the presence of the *NRAS^G12D^* mutation. The DEAPR approach to pathway analysis also revealed the importance of the FGF signaling pathway and the genes associated with it (*NCAM1*, *MMP9*, *SPP1*, *CDH2*, *JUN*, and *FGFR1*), and, indeed, when *NCAM1* levels were low, the *MMP9* levels were high and vice versa. The appearance of *SPP1* among the top three pathways for the *NRAS^G12D^*-specific pathway analysis suggests it is also a major player in the *NRAS^G12D^* phenotype.

Other mutant-specific expression patterns highlighted other potential therapeutic targets, such as *AKR1C1*, *AKR1C2*, and *AKR1C3* in the NRAS^G12V^ mutant, and calprotectin (*S100A8* and *S100A9*) in the *NRAS^G12D^* mutant. In squamous cell carcinoma, *AKR1C2* has already been shown to be a targetable oncogene [28]. Calprotectin’s role in AML and response to treatment is highly complicated and variable depending on the AML subtype, as is its role in cancer progression and the mechanisms involved, and it has been suggested as a potential therapeutic target [29].

Currently, they are no *NRAS*-mutant-specific drugs to treat hematological cancers because of the numerous challenges associated with this effort, while selective inhibitors of *KRAS^G12V^*, *KRAS^G12D^*, and *KRAS^G12C^* mutants are already being tested in solid tumors [30]. However, when a drug screen of 843 drugs was performed on six different *NRAS* G12 mutations in Ba/F3 cells, the *NRAS^G12D^* mutants were found to be more sensitive to *MEK* inhibition, while the *NRAS^G12V^* mutants were more sensitive to *Akt*, *EGFR*, *PLK*, *Src*, and *TGF*-β receptor inhibition [7]. Interestingly, these drugs inhibit the genes involved in cell cycle progression. The approach taken here peeled away the cell cycle genes commonly expressed in both *NRAS^G12D^* and *NRAS^G12V^* cells and exposed a potential secondary vulnerability associated with the type of mutant, in particular the *NRAS^G12D^*-specific expression of *MMP9* and *SPP1*, and the *NRAS^G12V^*-specific expression of *AKR1C2* and *AKR1C3*.

## 5. Conclusions

Since the introduction of RNA-sequencing, improvements in RNA isolation techniques, RNA-sequencing devices, mapping software, and reference genomes have elevated the accuracy of expression levels, thus providing laboratory researchers with the opportunity to look for consistent and reproducible differences among experimental groups for protein-coding genes in a manner comparable to quantitative PCR [23]. The DEAPR logic was developed to effectively interrogate carefully controlled laboratory experiments where the low number of samples in each experimental group (typically *n* = 3) is too underpowered for most DE software programs [10].

In this instance, DEAPR was used to evaluate the effects of removing an *NRAS^G12D^* mutation from THP-1 cells and replacing it with an *NRAS^G12V^* mutation [8]. The results showed a remarkable change within the immune response genes and, most specifically, with the calprotectin genes (*S100A8* and *S100A9*), which were upregulated in association with the G12D mutant and downregulated with the G12V mutation. These results suggest different treatment options should be considered based on the type of *NRAS* mutation and the distinct leukemia biology based on the type of *NRAS* missense mutation that is present.

## Figures and Tables

**Table 1:** A summary of the differences between DEAPR and DESeq2 when identifying DE genes and ranking their importance.

Description of Differences	DEAPR	DESeq2
Unit of measurement	FPKM	NGCs
Inherent bias toward longer genes	No	Yes
Introduces large distortions in fold change (and ranking) in cases where a gene has no expression in at least one but not all samples in an experimental group	No	Yes
Selects genes failing to demonstrate reproducibility (having overlapping expression values between experimental groups)	No	Yes
Provides a strategy for excluding genes with low expression that fall into the normal variance found within technical replicates	Yes	No
Includes a weighting strategy to provide increased importance to genes expressed at higher levels, thereby reflecting the ability of those genes to more successfully compete for translation initiation machinery	Yes	No
Easily selects protein-coding genes	Yes	No
Provides an easy method to rank pathways based on gene ranking	Yes	No

FPKM = Fragments per kilobase of transcript per million mapped reads (as generated by StringTie); NGCs = normalized gene counts (as generated by DESeq2 from StringTie gene counts).

## Data Availability

RNA-Sequencing data that support the findings of this study were deposited in the Gene Expression Omnibus with the following primary accession code: GSE115911.

## Supplementary Materials

The following supporting information can be downloaded at: https://www.mdpi.com/article/10.3390/cancers17030467/s1. Supplementary Methods and Figures: Figure S1: Number of DE genes selected within each gene length group, Figure S2: Expression levels in FPKMs of the top 50 DE genes, Figure S3: The FPKM levels for *MMP9* for each sample organized by experimental set, Figure S4: Ranking of DESeq2 DE genes as compared to the top 100 ranked DE genes from DEAPR, Figure S5: The FPKM levels for *SPP1* for each sample organized by experimental set. Supplementary Tables: Table S1: Variability analysis of protein coding genes, Table S2: Variability analysis of non-protein coding genes, Table S3: Results of DEAPR Comparison 3 (Comp3), based on FPKMs, DE genes sensitive to 1 ug of dox, Table S4: Results of DEAPR Comparison 8 (Comp8), based on FPKMs, DE genes sensitive to 10 ug of dox, Table S5: Results of DEAPR Comparison 1 (Comp1), based on FPKMs, DE genes found in *NRAS-G12D,* Table S6: Results of DEAPR Comparison 2 (Comp2), based on FPKMs, DE genes found in *NRAS-G12V,* Table S7: Results of DEAPR Comparison 4 (Comp4) - DE genes common to *NRAS-G12D* (Comp1) and *NRAS-G12V* (Comp2), based on FPKMs, Table S8: Results of DEAPR Comparison 5 (Comp5), based on FPKMs, DE genes specific to *NRAS-G12D,* Table S9: Results of DEAPR Comparison 6 (Comp6), based on FPKMs, DE genes specific to *NRAS-G12V,* Table S10: Results of DEAPR Comparison 7 (Comp7), based on FPKMs, DE gene with excessive *NRAS-G12V,* Table S11: Top 500 protein-coding DE genes identified by DESeq2 and compared to DEAPR generated list, Table S12: Top 500 protein-coding DE genes identified by DEAPR and compared to DESeq2 generated list2, Table S13: FPKM values of top 20 protein-coding DE genes for Comp1 as identified by DEAPR and compared to DESeq2 rank3. Table S14: Normalized gene counts of top 20 protein-coding DE genes for Comp1 as identified by DEAPR and compared to DESeq2 rank, Table S15: FPKM values of top 20 protein-coding DE genes for Comp1 as identified by DESeq2 and compared to DEAPR rank, Table S16: Normalized gene counts of top 20 protein-coding DE genes for Comp1 as identified by DESeq2 and compared to DEAPR rank, Table S17: Side by side comparison of top 500 DEAPR genes to the top 500 DESeq2 genes, sorted by the DESeq2 ranking, Table S18: Top 10 pathways identified by DEAPR modified GA Pathway analysis for Comp1 and Comp2, Table S19: Results of DEAPR modified GeneAnalytics pathway analysis for *NRAS-G12D* DE genes (Comp1), Table S20: Results of DEAPR modified GeneAnalytics pathway analysis for *NRAS-G12V* DE genes (Comp2), Table S21: Top 10 pathways identified by DEAPR modified GA Pathway analysis for Comp5 and Comp6, Table S22: Results of DEAPR modified GeneAnalytics pathway analysis for *NRAS-G12D*-specific DE genes (Comp5), Table S23: Results of DEAPR modified GeneAnalytics pathway analysis for *NRAS-G12V*-specific DE genes (Comp6), Table S24: Genes differentially expressed in both Comp2 and Comp7.
