# Supplementary material for: DEAPR: Differential Expression and Pathway Ranking Tool Demonstrates NRASG12V and NRASG12D Mutations Have Differing Effects in THP-1 Cells
Source: Cancers (Basel). 2025 Jan 30;17(3):467. doi: 10.3390/cancers17030467 (PMC11816133; doi:10.3390/cancers17030467)
Supplement: Supplementary file 1 [file cancers-17-00467-s001.zip › Supplementary Methods and Figures.pdf]

DEAPR: Differential Expression and Pathway Ranking tool demonstrates *NRAS*^{G12V} and *NRAS*^{G12D} mutations have differing effects in THP-1 cells

Susan K. Rathe[1*], Jeremy P. White[1], Zohar Sachs[1,4], David A. Largaespada[1,2,3]

[1]Masonic Cancer Center, University of Minnesota, Minneapolis, MN, USA
[2]Center for Genome Engineering, University of Minnesota, Minneapolis, MN, USA
[3]Department of Pediatrics, University of Minnesota, Minneapolis, MN, USA
[4]Division of Hematology, Oncology, and Transplantation, Department of Medicine, University of Minnesota, Minneapolis, MN, USA

Supplementary Methods and Figures

Guide for the use of Differential Expression and Pathway Ranking (DEAPR)

The ***deapr*** and ***pathway*** programs were written in python. Python is a free programming language available for download at https://www.python.org/.

The ***deapr*** program requires 2 input files: a table of FPKM values and a table of genes with the associated gene types. The FPKM table was built by first mapping the raw fastq files using HISAT2 with an Ensembl gtf (reference transcriptome file) and specifying a splice penalty (--pen-noncansplice) of 6 and an output format recognizable by StringTie (--downstream-transcriptome-assembly). The resulting bam file was used as input to StringTie, which was run with the parameter to output a gene abundance estimation file (-A).

The Ensembl ID, Gene Name, and FPKM values from the Gene Abundance files for each sample were merged into a single table. The first column contained the Ensembl ID with a header specifying Ensembl ID. The second column contained the gene name with the header Gene Name. The remaining columns were the FPKMs for each sample with a simple unique identifier for each sample in the header line. In this case, S1-S18 were used.

A gene type table was downloaded from Ensembl BioMart by selecting the Ensembl Genes 109 (dataset) and Human gene (GRCh38.p13). Selected attributes included Gene stable ID, Gene name, and Gene type and then downloaded. In Excel, a tab-delimited text file was made from the downloaded file with just the Gene stable ID (first column) and Gene type (second column). Only the "protein_coding" genes were kept and the headers removed. The ***deapr*** program specifically selects genes with a gene type of either "protein coding" or "protein_coding".

To execute ***deapr***: the FPKM table, gene type table, and the ***deapr*** program were copied into a PC folder, and the following commands were executed:

For comp1:

python deapr.py Raw_data.txt Protein_coding_genes.txt S10,S11,S12 "First group" S1,S2,S3 "Second group" --output comp1.csv

For comp2:

python deapr.py Raw_data.txt Protein_coding_genes.txt S10,S11,S12 "First group" S13,S14,S15 "Second group" --output comp2.csv

For comp3:

python deapr.py Raw_data.txt Protein_coding_genes.txt S1,S2,S3 "First group" S4,S5,S6 "Second group" --output comp3.csv

For comp7:

python deapr.py Raw_data.txt Protein_coding_genes.txt S13,S14,S15 "First group" S16,S17,S18 "Second group" --output comp7.csv

For comp8:

python deapr.py Raw_data.txt Protein_coding_genes.txt S4,S5,S6 "First group" S7,S8,S9 "Second group" --output comp8.csv

The top 400 DEAPR genes were copied to a tab delimited text file and used as input to GeneAnalytics (GA). After execution the GA output was downloaded and opened in Excel. The list of pathways was copied from the Pathway worksheet (the first 5 columns from the Results section (including the 1 line header) and placed into a tab delimited text file.

The ***pathway*** program was executed using the output from the deapr program and the tab delimited text file copied from the GA Pathway worksheet. An example of the ***pathway*** command:

python pathway.py comp1.csv comp1_GA_output.txt --output comp1_pathways.csv

The ***pathway*** program also recognizes when GA automatically changes a gene name, by outputting a warning message: Missed gene. When this happens, the new gene name can be queried in GeneCards to find previous gene names. If one of the previous gene names is found in the ***deapr*** output file, it can be changed to the new gene name and then the ***pathway*** logic can be run again.

The impact of gene length when ranking genes by DEAPR and DESeq2

To determine if there was a gene length bias experienced, by either the DEAPR method using FPKMs or the DESeq2 method using normalized gene counts, the length of the longest primary transcript for each protein coding gene was placed in a table and grouped by length with ~ 4450 genes in each group.

	Transcript Length	
Group	From	To
1	76	1497
2	1498	2329
3	2330	3438
4	3440	5239
5	5240	or more

Next the top 500 ranked DE genes from both the DEAPR and DESeq2 methods were evaluated by the length of the primary gene transcript. Approximated 2/3rds of the genes were common to both methods and demonstrated an even distribution by gene length (Figure S1A), as did the genes that were only found in the top 500 of the DEAPR DE gene list (Figure S1B). However, the genes that were only found in the DESeq2 list were highly biased toward longer genes (Figure S1C).

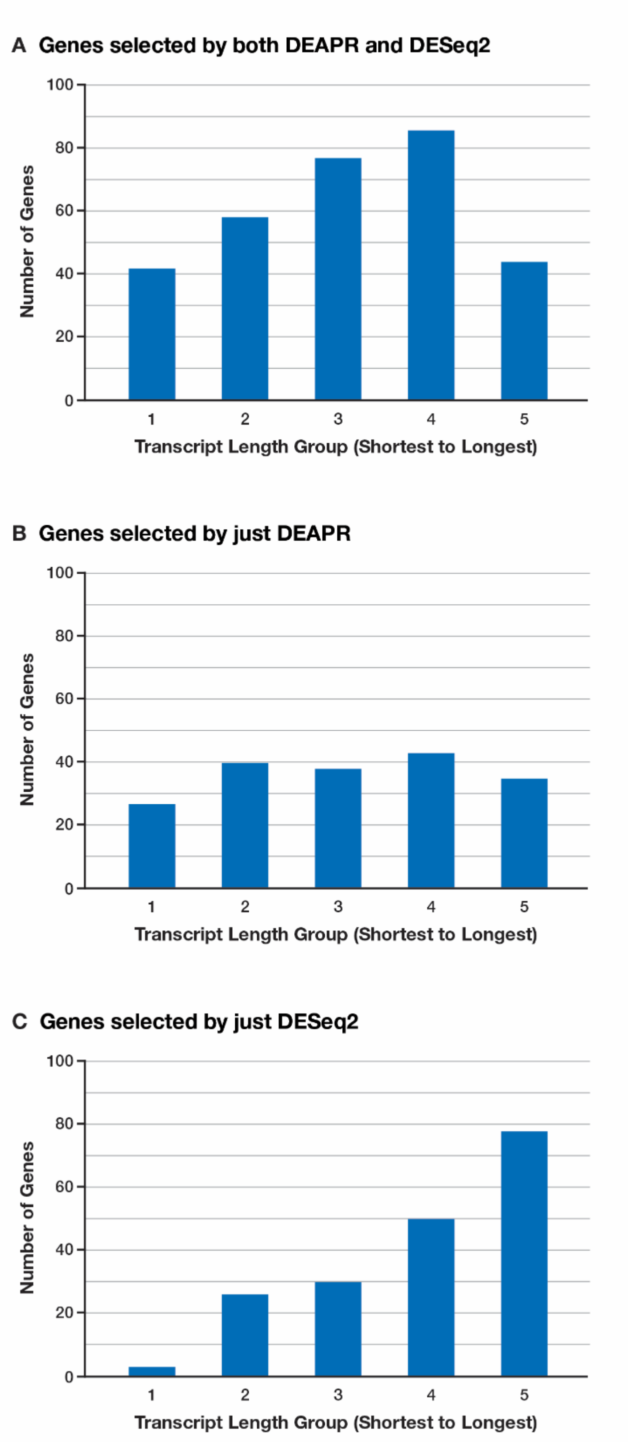

Figure S1: Number of DE genes selected within each gene length group. A) DE genes selected in the top 500 by both DEAPR and DESeq2, B) DE genes selected in the top 500 by just DEAPR, and C) DE genes selected in the top 500 by just DESeq2.

DESeq2 demonstrates a bias toward genes expressed at lower levels

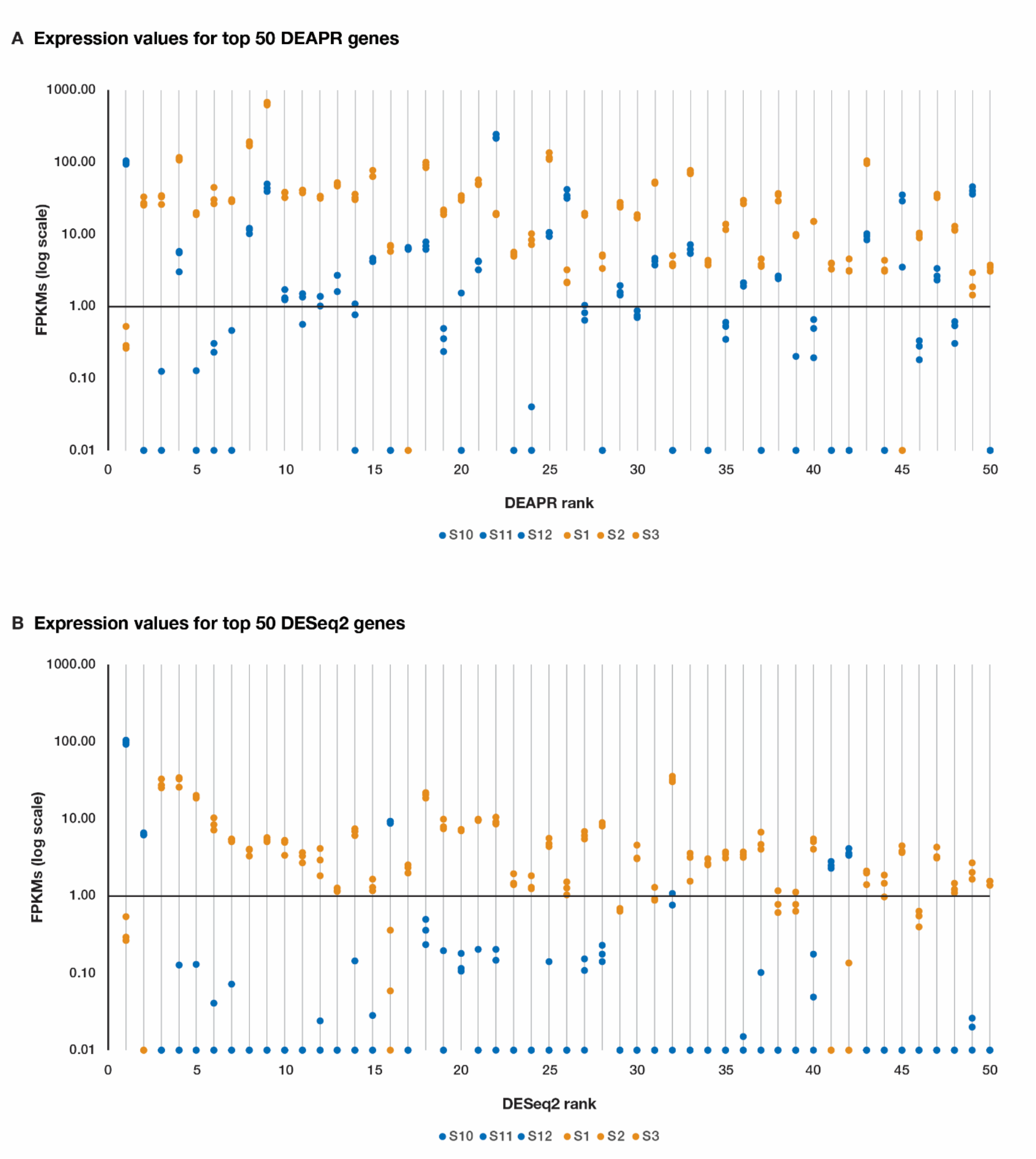

Figure S2: Expression levels in FPKMs of the top 50 DE gene identified by A) DEAPR and B) DESeq2.

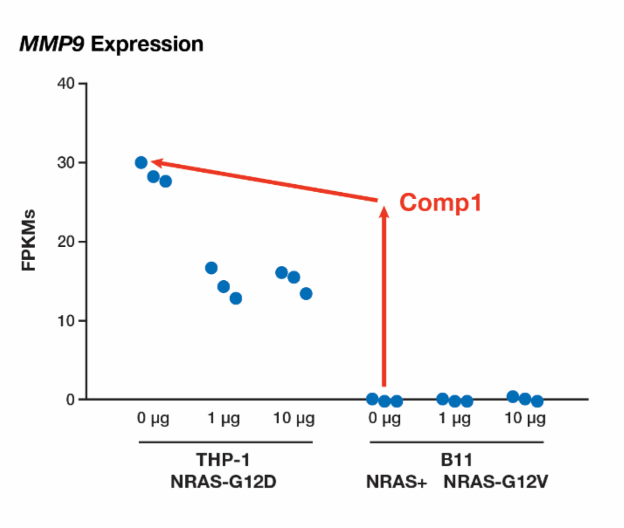

Figure S3: The FPKM levels for *MMP9* for each sample organized by experimental set. The red arrows show the Comp1 comparison of the no NRAS mutant control (B11 0 µg dox) to the $NRAS^{G12D}$ mutant (THP-1 0 µg dox), indicating MMP9 expression is specific to the $NRAS^{G12D}$ mutant. (n=3 in each set of samples)

Adding 1 to all the gene counts results in a more logical ranking of some genes by DESeq2 but also greatly reduces the importance of many other genes

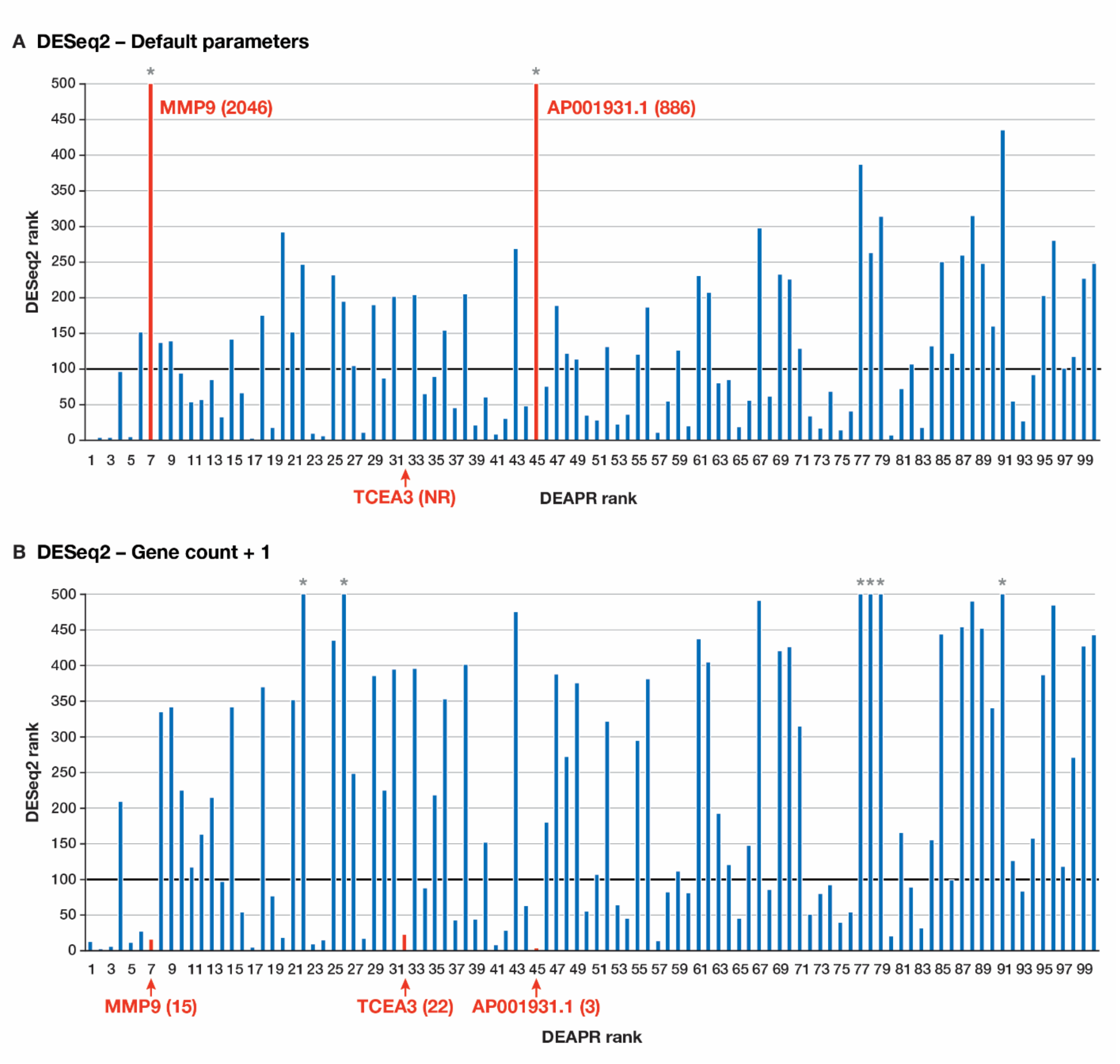

Figure S4: Ranking of DESeq2 DE genes as compared to the top 100 ranked DE genes from DEAPR: A) using unmodified gene counts from StringTie and B) adding 1 gene count to each gene count. Genes in red show their DESeq2 ranking in parenthesis (NR=not ranked). Asterisks (*) indicate the DESeq2 rank was above 500.

Logic behind selecting the top 400 genes for pathway analysis

The selection of the top 400 genes from the DEAPR output was not arbitrary, but it may not be applicable for every project. For example, when evaluating the *NRAS*^{G12D} specific list of DE genes, the answers provided from GeneAnalytics (GA) will vary depending on the number of genes used:

**Pathway analysis of RNAS-G12D specific genes (Comp5)**		**Pathway ranking strategy**						
		**DEAPR**	**GeneAnalytics**					
**SuperPath Name**	**Path Ttl**	**top 400**	**top 100**	**top 200**	**top 400**	**top 800**	**top 1200**	**all 1456**
Mesenchymal Stem Cells and Lineage-specific…	95	1	2	1	4	7		
Vitamin D Receptor Pathway	185	2		3	1	6		
FGF Signaling Pathway	54	3	3	4	15			
Innate Immune System	2024	4		8	2	1	1	1
Signal Transduction	2591	5	20	2	3	2	2	2
ERK Signaling	1185	6	7	6	6	4	20	
PAK Pathway	686	7		10	5	15		18
Extracellular Matrix Organization	300	8	18	13	7			
Regulation of Nuclear Beta Catenin Signaling …	72	9	6	7	13			
Validated Transcriptional Targets of AP1 …	35	10		16	16			
Blood-Brain Barrier and Immune Cell …	104	11			9	5	21	
Integrin Pathway	570	12	1	5	11	14		
Lung Fibrosis	63	13	4	15	18			
Cytoskeleton Remodeling Regulation of Actin ,,,	187	14	5	11	14			
Cell Adhesion_Cell-matrix Glycoconjugates	39	15	14	12	17			
Malignant Pleural Mesothelioma	409	16		9	10	10	11	14
Hair Follicle Development: Organogenesis…	25	17			22			
Adhesion	123	18		20	26		17	20
Splicing Factor NOVA Regulated Synaptic …	39	19			8			
Interleukin-10 Signaling	47	20	8		28			

Number of genes input to GeneAnalytics
Top ranked pathways by GeneAnalytics

In this example, the pathway program was used to prioritize the output from the GA output when the top 400 genes were used. It resulted in the FGF Signaling Pathway being ranked 3rd, when it was ranked as 15th by GA, and not included in GA's top category of pathways (highlighted in green). If the top 800 genes had been used, the FGF Signaling Pathway would not have been identified at all, as was the case with all the other pathways that had Pathway Totals of 72 or less. This demonstrates the bias of the GA algorithm toward the larger pathways, when the list of DE genes is quite large. And yet, using less than the 400 limit runs the risk of ignoring some of the larger pathways altogether.

As a rule of thumb, start by eliminating the bottom 20% of the genes on the DEAPR list, since this is where the greatest percentage of false positives will be located. If that leaves more than 400 genes, then start with 400 and then run a couple of test cases in GA with smaller or larger number of genes to see if there may be a bias in the samples being studied.

In this case, the top 3 pathways in the DEAPR top 400 gene analysis were selected because of the high DEAPR rankings for the following genes:

**FGF Signaling Pathway**	Rank
NCAM1	1
MMP9	6
SPP1	14
CDH2	15
JUN	79
FGFR1	131

**Vitamin D Receptor Pathway**	
ORM2	12
SPP1	14
CD9	16
IGFBP3	34
SLC8A1	42
TPM1	104
S100A9	109
SFRP1	122
THBD	167
S100A8	180
SALL4	217
GADD45A	248
ALOX5	322
LRP5	356
NRIP1	378

**Mesenchymal Stem Cells and Lineage-specific**	
NCAM1	1
LPL	10
SPP1	14
CDH2	15
PPARG	26
IGFBP3	34
VCAN	67
WNT7B	119
TNNI3	210
KIT	216

Interestingly *SPP1* (osteopontin) with the DEAPR ranking of 14th was present in all 3 pathways, and it was $NRAS^{G12D}$ specific (Figure S4). Since *SPP1* was found associated with other highly ranked DE genes in multiple pathways, it should be explored further as a major player in the $NRAS^{G12D}$ phenotype.

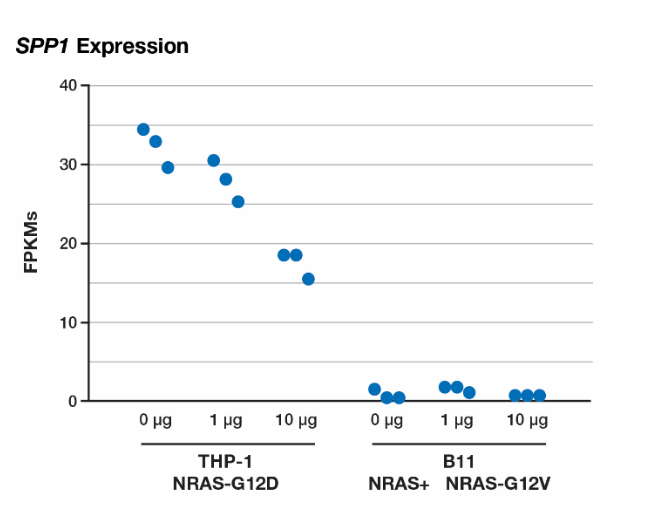

Figure S5: The FPKM levels for *SPP1* for each sample organized by experimental set (n=3 in each set of samples).
